# Are We Ready for Proactive Therapeutic Drug Monitoring of Anti-TNF to Optimize Care of Patients With Inflammatory Bowel Disease?

**DOI:** 10.1093/crocol/otz056

**Published:** 2020-01-24

**Authors:** Aline Charabaty

**Affiliations:** 1 Department of Medicine, Johns Hopkins School of Medicine, Washington, DC; 2 Division of Gastroenterology, Sibley Memorial Hospital, Johns Hopkins University, Washington, DC; 3 IBD Center, Johns Hopkins Sibley Memorial Hospital, Washington, DC

The goal of therapeutic drug monitoring (TDM) in inflammatory bowel disease (IBD) is to assess drug level and the presence/absence of antidrug antibodies at a certain point in the treatment timeline to adjust therapy to overcome (reactive TDM) or prevent (proactive TDM) loss of response. In 2017, the AGA Institute published guidelines recommending reactive TDM in patients with partial response or loss of response to anti-TNF and subsequently optimizing or switching therapy within or outside drug class based on TDM results. No recommendations were made for proactive TDM, that is, assessing drug levels in patients who are in clinical remission and adjusting drug dose and/or frequency to reach and maintain a “therapeutic” drug level. However, we now know that low drug levels increase the risk of developing antidrug antibodies, which can lead to loss of response, and a reactive TDM and anti-TNF dose adjustment at that point might be too late to salvage the drug and recapture response. We also know that the best therapeutic effect is achieved with the first biologic the patient uses, and response to subsequent biologics is decreased in patients who have been previously exposed to anti-TNF. So it is crucial to get the right amount of drug in the patient early on, to optimize chances of remission, and to avoid loss of response over time to the first biologic. On the other hand, one can argue that it is even more important to optimize drug levels and chances of response in a patient who has already failed or lost response to one or more biologic to avoid running out of treatment options.

In this issue of CC360, Queiroz et al offer a comprehensive review of the available data on the association between anti-TNF drug levels, antidrug antibodies and treatment response, the timing of proactive TDM during the course of therapy, and the anti-TNF drug threshold after induction and during maintenance associated with optimized IBD outcome.

For those that argue that TDM adds to the cost of IBD care, cost-effective analyses concluded that TDM-based dosing is more cost-effective than empiric dose escalation in IBD patients losing response or with partial response to anti-TNF. In addition, using TDM and adjusting infliximab dosing to achieve a therapeutic level might obviate the need for combination therapy with thiopurines to optimize treatment response or durability.^1, 2^ Avoiding thiopurines reduces the cost associated with additional drug and monitoring as well as the risk of infection and lymphoma associated with combination therapy.

Several retrospective studies showed that higher drug levels early after induction and during maintenance were associated with clinical, biologic, and endoscopic remission. A post hoc analysis of TAILORIX (a randomized controlled trial that compared infliximab dose escalation based on drug level and elevated biomarkers to dose escalation based on symptoms alone in Crohn patients) showed that infliximab level > 23 mcg/mL at week 2 and >10 mcg/mL at week 6 were associated with endoscopic remission as early as week 12.^3^ Compared with reactive TDM, proactive TDM in patients in remission after induction with infliximab was associated with less antidrug antibodies formation and infusion reactions, increased drug durability, and decreased IBD-related hospitalization and surgeries.^4^

However, most studies showing an association between high drug level and disease remission are retrospective and correlative in nature. Another argument that has been made against TDM is the following: is it the high drug level that puts the disease in remission, or is the disease that is responsive to the therapy that leads to a decreased drug clearance from a healed bowel and higher serum drug levels (and patients with more severe disease or disease that does not respond to therapy, have a higher drug clearance, and have low serum drug levels). A recent prospective observational UK study, the PANTS study^5^ showed that infliximab levels > 7 mcg/mL and adalimumab drug levels > 12 mcg/mL at week 14 were associated with clinical remission at weeks 14 and 54.^5^ The PAILOT study (Pediatric Crohns Disease Adalimumab-Level-Based Optimization Treatment) is the first randomized controlled trial that showed better Crohn outcome in children undergoing proactive TDM versus reactive TDM. Children with Crohn disease who responded to adalimumab induction were randomized to proactive optimization of therapy versus reactive TDM. In the proactive arm, adalimumab dose adjustments were made to reach a trough drug level between 5 and 10 mg/mL. The primary end point, which was sustained corticosteroid-free clinical remission at all visit (from week 8 through week 72), was significantly higher in the proactive TDM group versus reactive group (82% vs 48%, *P* = 0.002).^6^

Although the data reviewed here argue for the usefulness of proactive TDM, there are several questions that remain to be answered before proactive TDM becomes standard of care for our patients with IBD on anti-TNF. It is not clear how often drug levels need to be checked after the initial optimization and what target trough levels are needed during maintenance beyond a year in patients with sustained clinical and/or endoscopic remission. Target drug levels might be different based on disease behavior, severity, and phenotype. For example, some data suggest that higher infliximab drug level might be needed for perianal fistula healing or that a lower trough level of infliximab could be enough to prevent Crohn disease recurrence postoperatively.^7^ Finally, we need data to assess whether the presence of a high drug trough level during proactive TDM would allow for safe dose/frequency de-escalation in patients in endoscopic healing. Overall, proactive TDM needs to be further evaluated with large randomized controlled trials before we can offer an effective and personalized approach to TDM to our IBD patients.
